# No hints at glyphosate-induced ruminal dysbiosis in cows

**DOI:** 10.1038/s41522-021-00198-4

**Published:** 2021-03-25

**Authors:** Fabian Billenkamp, Karina Schnabel, Liane Hüther, Jana Frahm, Dirk von Soosten, Ulrich Meyer, Dirk Höper, Martin Beer, Christian Seyboldt, Heinrich Neubauer, Sven Dänicke

**Affiliations:** 1 Institute of Animal Nutrition, Federal Research Institute for Animal Health, Friedrich-Loeffler-Institute, Brunswick, Germany; 2 Institute of Diagnostic Virology, Federal Research Institute for Animal Health, Friedrich-Loeffler-Institute, Greifswald-Riems, Germany; 3 Institute of Bacterial Infections and Zoonoses, Federal Research Institute for Animal Health, Friedrich-Loeffler-Institute, Jena, Germany

**Keywords:** Microbiome, Pathogens

## Abstract

Glyphosate-based herbicides are among the most used non-selective herbicides worldwide and inhibit synthesis of aromatic amino acids in plants, bacteria, and fungi. Given the broad usage, controversies concerning potential effects of glyphosate on health and especially on gut microbiomes arose. For cattle, it has been proposed based on in vitro data that glyphosate has detrimental effects on the ruminal microbiome, which manifest as a specific inhibition of bacteria involved in fiber degradation and as an enrichment of specific pathogens. In the present study, glyphosate effects on the ruminal microbiome were analyzed in vivo using glyphosate contaminated feedstuffs with strong differences in dietary fiber and dietary energy content in order to reproduce the proposed detrimental glyphosate effects on the rumen microbiome. While significant impact of dietary factors on the ruminal microbiome and its products are pointed out, no adverse glyphosate effects on ruminal microbiome composition, diversity, and microbial metabolites are observed.

## Introduction

Glyphosate is one of the most used active substances in common non-selective herbicides worldwide and was considered an advantageous herbicide until the first glyphosate-resistant weeds appeared^1^. Supported by introduction of glyphosate-resistant genetically modified crops in the 1990s, glyphosate applications and concerns about applications steadily increased^2–4^. Beneath concerns about human exposure to glyphosate, especially the continuous exposure of livestock to glyphosate is under observation. Usage of glyphosate-based herbicides has been common in Germany for an extended period of time despite increasing strict EU regulations and more than 25% of utilized agricultural areas in Germany had an application of glyphosate-based herbicides in 2009^5^. However, soybean meal from glyphosate-resistant soybeans is considered the main source of glyphosate contaminations in German cattle feed, while residues from local crops are negligible^6^. Since glyphosate contaminations in feed production and consequently also ingestion by livestock animals are common, potential exposure and effects of glyphosate on livestock have been evaluated frequently^7–9^. For dairy cows, the main proportion of consumed glyphosate, which ranges in average daily exposures from 0.08 to 6.7 mg per cow, is excreted via feces and urine, while only negligible amounts of glyphosate are excreted via milk^6^. Health and performance of dairy cows did not respond to glyphosate contaminations in feed^10–12^. Glyphosate inhibits synthesis of aromatic amino acids through inhibition 5-enolpyruvyl-shikimic acid-3-phosphate synthase in plants and susceptible bacteria^13–15^. Accordingly, a potential impact of glyphosate on microbiota in digestive organs, which are exposed on regular basis and directly linked to animal health, is in the focus of interest. Recommended glyphosate application has only limited effects on soil microbiomes and different microbial degradation pathways for glyphosate are known^16–18^. It was also pointed out that glyphosate has a limited short-term effect on gut microbiota upon ingestion due to sufficient amino acid supply in the gastrointestinal tract^19^. Rumen microbial composition is associated with feed composition, feed efficiency phenotypes, and performance effects, while microbiome disturbances are considered a basis for detrimental effects like pathogen enrichment^20–23^. In this context variations of concentrate feed proportion (CFP) and dietary fiber content are of special interest, since the chemical composition of diets defines the ruminal fermentative pattern and especially high CFP can lead to adverse effects including subacute ruminal acidosis and ruminal dysbiosis, which is characterized by strong shifts in microbiome composition^24–26^. Different studies analyzed a potential introduction of disturbances to the ruminal microbiome by glyphosate in vitro in the context of high CFP challenges and varying dietary fiber contents. The results were conflicting and ranged from no significant glyphosate effects in mixed cultures to proposing an enrichment of *Clostridium* (*C*.) *botulinum* and an increased neurotoxin gene expression^27–31^. Subsequently, such enrichment of *C. botulinum*, coupled to increased neurotoxin gene expression in the gut of dairy cows, has been controversially discussed in the context of a highly disputed chronic form of botulism^32–36^. However, data concerning the effects of glyphosate in the context of dietary challenges on the ruminal microbiome and putative consequences for animal health from in vivo trials in “real-life” scenarios is still limited.

To address the lack of knowledge about the influences of glyphosate on dairy cows in general and in order to evaluate discrepancies between the different studies, a broad general health screening of dairy cows under realistic housing conditions with different CFP and dietary fiber content in feedstuffs was conducted. Within this screening, influences of glyphosate on animal performance, liver histology, liver gene expression, and functionality of blood cells were tested and occurrence of significant effects was rejected^10–12^. To achieve this, cows were assigned to 4 groups receiving diets with a high CFP (60%, HC) or a low CFP (30%, LC) and with (GLY, glyphosate exposure per cow: >73 mg d^−1^; >112 µg kg(bodyweight)^−1^ d^−1^) or without (CON, glyphosate exposure per cow: <1 mg d^−1^; <1.3 µg kg(bodyweight)^−1^ d^−1^) glyphosate contaminations^10^. Next to the detailed screening of animal health, especially the question whether glyphosate detrimentally influences the ruminal microbiome in an interactive manner with the dietary composition was of interest. To address this question, rumen fluid samples were collected throughout the trial and subjected to 16S rDNA genotyping, analyses of bacterial endotoxin (lipopolysaccharide = LPS) content, and microbial fermentation products. Finally, a screening for abundance *C. botulinum* neurotoxin (BoNT) genes was conducted at the beginning and at the end of the trial in order to address the question of whether glyphosate influences a putative enrichment of this pathogen in the context of challenging CFP and varying amounts of dietary fiber.

## Results

### Analysis of microbial compounds and products in the rumen

**Fig. 1:**
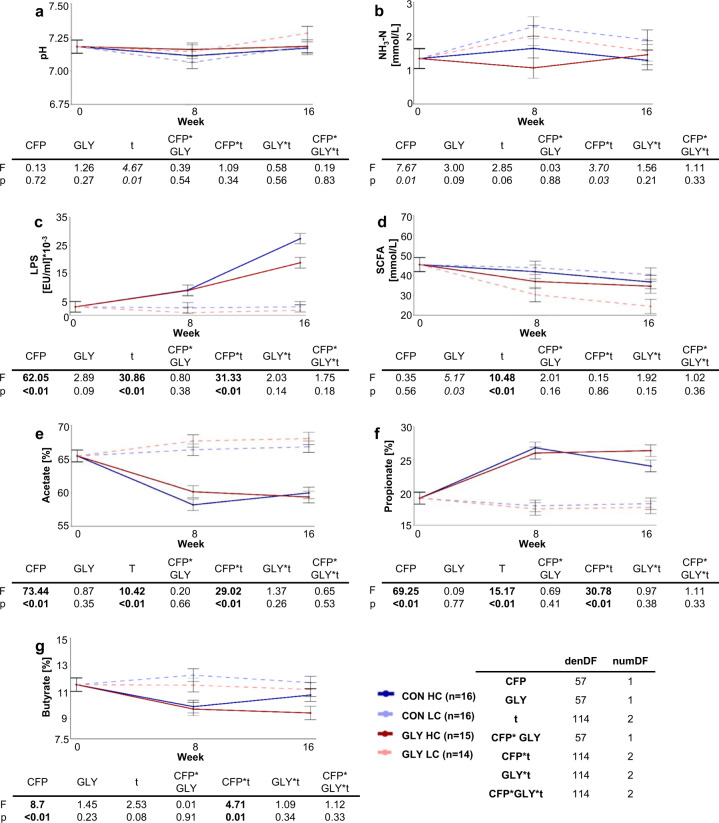
Influences of concentrate feed proportion and glyphosate on ruminal parameters. Influences of concentrate feed proportion (CFP) and glyphosate on ruminal pH (**a**), ruminal ammonia (NH_3_-N) concentration (**b**), ruminal lipopolysaccharide (LPS) concentrations (**c**), and total ruminal short-chain fatty acid (SCFA) concentrations (**d**). Furthermore, proportions of the major SCFAs acetate (**e**), propionate (**f**), and butyrate (**g**) are depicted. Displayed are group lsmeans together with their respective standard error and the results of one-tailed ANOVAs of linear mixed effects models with fixed factors CFP, glyphosate exposure (GLY), time (*t*), and the interactions for the individual parameters. Number of individuals (*n*) and degrees of freedom were identical for all parameters and are displayed in the figure. Blue: control diet (CON), red: glyphosate contaminated diet (GLY), dark solid lines: high CFP (HC), light dashed lines: low CFP (LC), denDF: denominator degrees of freedom, numDF: numerator degrees of freedom, values in bold: *p* < 0.01, values in italics: *p* < 0.05.

Throughout the trial, ruminal pH was altered, which was reflected by a significant time effect (p_t_ < 0.01, Fig. 1a). Ruminal ammonia was significantly influenced by CFP (p_CFP_ < 0.05) and changes over time differed between groups with different CFPs (p_CFP*t_ < 0.05, Fig. 1b). The ruminal LPS concentration was significantly influenced by time (p_t_ < 0.01) and CFP (p_CFP_<0.01). Changes in LPS concentrations over time also clearly differed between groups with different CFPs (p_CFP*t_ < 0.01; Fig. 1c). All three parameters were not significantly influenced by glyphosate effects. Total short-chain fatty acid (SCFA) concentrations in the rumen were significantly varying over time (p_t_ < 0.01) and differed significantly between CON and GLY groups (p_GLY_ < 0.05, Fig. 1d), while no significant differences in variations over time were observed between experimental groups. For the relative proportions of the major SCFAs acetate, propionate, and butyrate, a significant effect of CFP (p_CFP_ < 0.01) was observable and changes over time significantly differed between groups with different CFPs (p_CFP*t_ < 0.01; Fig. 1e–g). Furthermore, a significant alteration over time on the proportions of acetate and propionate was observable (p_t_ < 0.01). In contrast to this, no effect of glyphosate on major SCFA proportions was observable. Similarly, the low relative proportions of valerate and the branched-chain SCFA isobutyrate were influenced by CFP (p_CFP_ < 0.01) and time (p_t_ < 0.01) changes in proportions over time differed between groups with different CFPs (p_CFP*t_ < 0.01, Supplementary Fig. 1a, b). Finally, for the branched-chain SCFA isovalerate, significant influences of glyphosate (p_GLY_ < 0.05) and time (p_t_ < 0.01) were observed, while alterations over time differed between GLY and CON groups (p_GLY*t_ < 0.05; Supplementary Fig. 1c).

### Sequencing and data preparation

To evaluate the influences of GLY and CFP on the ruminal microbiome, a 16S rDNA genotyping was conducted. The individual sequencing libraries had an average size of 178,330 reads. After quality filtering an average of 117,601 reads per library remained. Normalization for the size of the smallest library retained 28% of the total initially sequenced reads and 18,518 unique sequences, which were represented as 50,546 remaining reads per individual library (see Supplementary Table 1).

### Rumen alpha-diversity

**Fig. 2:**
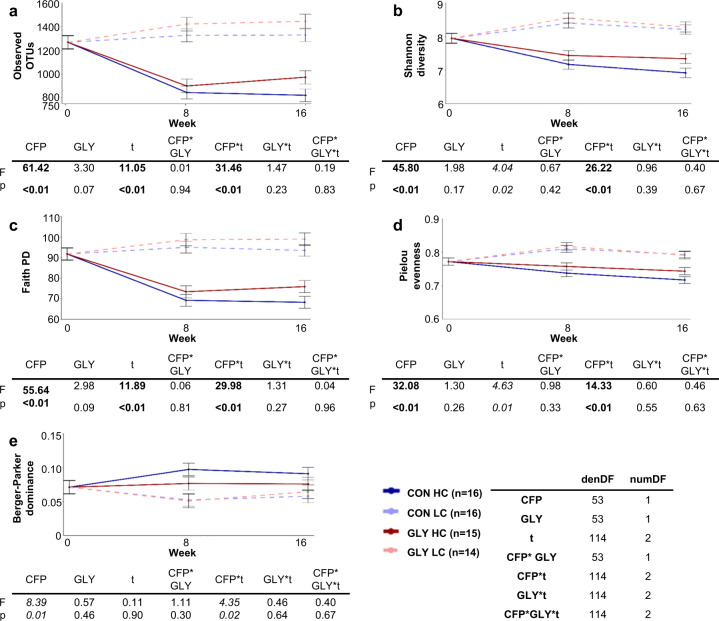
Influences of concentrate feed proportion and glyphosate on rumen microbial alpha-diversity. Influences of concentrate feed proportion (CFP) and glyphosate on observed operational taxonomic units (OTUs) in the rumen (**a**), the Shannon index of the rumen microbial community (**b**), and rumen phylogenetic diversity displayed as Faith’s phylogenetic diversity (**c**). In addition, evenness of the rumen microbial community as Pielou index (**d**) and dominance in the microbial displayed by Berger–Parker index (**e**) are depicted. Displayed are group lsmeans together with their respective standard error and the results of one-tailed ANOVAs of linear mixed effects models with fixed factors CFP, glyphosate exposure (GLY), time (*t*), and the interactions for the individual parameters. Number of individuals (*n*) and degrees of freedom were identical for all parameters and are displayed in the figure. Blue: control diet (CON), red: glyphosate contaminated diet (GLY), dark solid lines: high CFP (HC), light dashed lines: low CFP (LC), denDF: denominator degrees of freedom, numDF: numerator degrees of freedom, values in bold: *p* < 0.01, values in italics: *p* < 0.05.

In order to compare animal-individual microbiome compositions, analyses of microbial alpha-diversity, evenness, and dominance were conducted. As measurements for diversity, the number of detected operational taxonomic units (OTUs), the Shannon index, and phylogenetic diversity based on Faith’s metric were significantly influenced by CFP (p_CFP_ < 0.01), time (p_t_ < 0.05), and changes over time significantly differed between the groups with different CFPs (p_CFP*t_ < 0.01; Fig. 2a–c). Pielou evenness was similarly influenced by CFP (p_CFP_ < 0.01), time (p_t_ < 0.05), and displayed different alterations for groups with different CFPs over time (p_CFP*t_ < 0.01; Fig. 2d). Berger–Parker dominance was influenced by CFP (p_CFP_ < 0.05) and variations over time differed between groups with different CFP (p_CFP*t_ < 0.05; Fig. 2e). Glyphosate did not influence any of these measures.

### Rumen beta-diversity

**Fig. 3:**
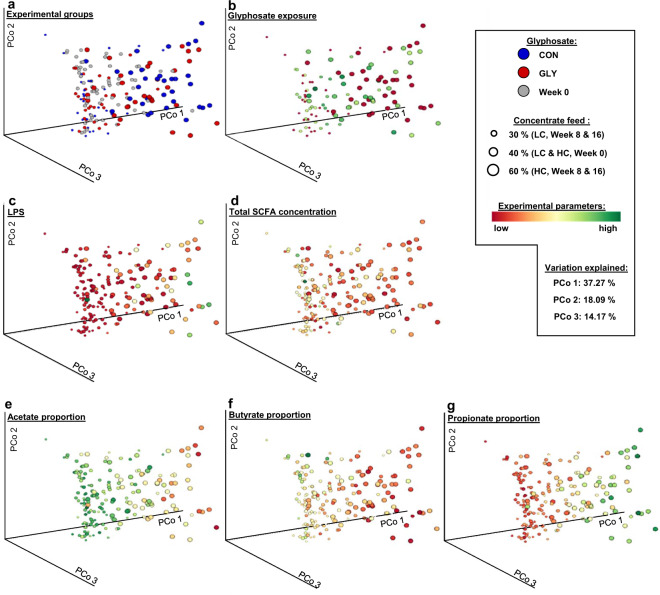
Principal Coordinate Analysis of the rumen microbial community based on weighted UniFrac distance. Displayed are different colorations of the identical principal coordinate analysis (PCoA) analysis of the rumen microbial community based on weighted UniFrac distance. Coloration in discrete scaling: **a** experimental groups. Colorations in continuous scaling: **b** glyphosate exposure in weeks 8 and 16, **c** concentration of total ruminal short-chain fatty acids (SCFAs), **d** concentration of ruminal LPS, **e**–**g** proportions of acetate, butyrate and propionate among ruminal SCFAs. CON: control group, GLY: group with glyphosate contaminated diet, Week 0: values before feeding CON or GLY diets, CFP: concentrate feed proportion, LC: low CFP, HC: high CFP, PCo: principal coordinate.

To assess whether major differences in microbial composition between animals were influenced by glyphosate exposure or different CFPs, a distance matrix based on weighted UniFrac distance was calculated and subjected to an Adonis test (Table 1), a permutational ANOVA (permANOVA, Supplementary Table 2), tested for homogeneity of multivariate dispersions (permdisp, Supplementary Table 2), as well as subjected to an analysis of similarities (anosim, Supplementary Table 2) using Qiime2^37^. In the Adonis test, significant influences of CFP (*p* < 0.01) and of an interaction between CFP and time (*p* = 0.01) on distance between samples were observed, while glyphosate and its interactions with other experimental factors did not significantly influence the distance between samples (Table 1). However, only a limited proportion of the distances between samples could be explained through CFP or its interaction with time (*R*² = 0.10 for CFP and *R*² = 0.05 for CFP*t). The observed influence of CFP on distance between samples is supported by the results from permANOVA, permdisp, and anosim. In permANOVA and anosim, differences between groups with different CFP were significantly higher than differences within groups (*p* < 0.01 for both tests, Supplementary Table 2), while differences between groups were not significantly influenced by glyphosate (*p* > 0.05, Supplementary Table 2). Significant effects caused through homogeneity of multivariate dispersions were not observed in permdisp (*p* > 0.05, Supplementary Table 2) for any of the significant group differences detected in permANOVA. In order to visualize distances in microbiome composition between the individual samples, a principal coordinate analysis (PCoA) based on the weighted UniFrac distance matrix was conducted. Assigning color and shape-size to experimental groups made apparent, that animals with different CFPs had bigger distances between each other than animals fed with the same CFP (Fig. 3a). Presence of glyphosate contaminations in the diet in contrast apparently did not influence the distance between samples (Fig. 3a). Coloration by average daily glyphosate exposure in weeks 8 and 16 of the experiment did not indicate clustering of samples based on the amount of exposure (Fig. 3b). Coloration based on total ruminal SCFA concentration resulted in a color gradient in the visualization, which indicated smaller distances of the ruminal microbiome of animals with similar concentrations of total SCFAs than of animals with different concentrations (Fig. 3c). Coloration by ruminal LPS concentration indicated, that the majority of animals had low ruminal LPS concentrations and that most animals with a high ruminal LPS concentration were located in one region in the PCoA (Fig. 3d). Comparison to the experimental groups (Fig. 3a) indicated, that high ruminal LPS content was coupled to a high CFP and independent of glyphosate exposure. When using colorations based on the major ruminal SCFA proportions, color gradients indicated, that the ruminal microbiomes of animals with similar proportions of SCFAs hat lower distances to each other than the ruminal microbiomes of animals with different proportions of SCFAs (Fig. 3e–g). A comparison to the experimental groups (Fig. 3a) indicated a glyphosate-independent CFP-dependency of these gradients.

**Table 1:** Adonis test for influence of concentrate feed proportion, glyphosate, and time on weighted UniFrac distances between microbial communities.

	Df	meanSqs	*R*^2^	*F*	*p*
CFP	1	0.87	0.10	17.1	<0.01
GLY	1	−0.71	−0.08	−13.9	1.00
*t*	2	0.04	<0.01	0.7	0.46
CFP*GLY	1	−0.22	−0.02	−4.3	0.87
CFP*t	2	0.46	0.05	9.1	0.01
GLY*t	2	−0.18	−0.02	−3.6	0.82
CFP*GLY*t	2	−0.35	−0.04	−7.0	0.99

*CFP* concentrate feed proportion, *GLY* glyphosate exposure, *t* time, *Df* degrees of freedom, *meanSqs* mean squares, *R*² partial *R*-squared.

### Taxonomic classification

**Fig. 4:**
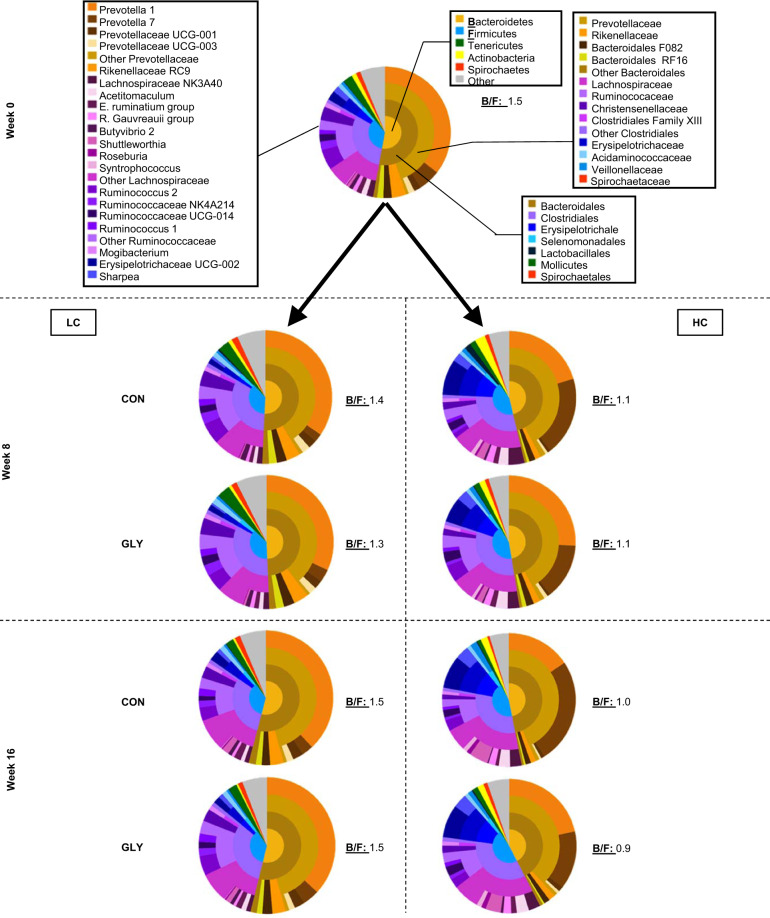
Taxonomic composition of the ruminal microbiome in response to varying concentrate feed proportion and glyphosate. Depicted are donut plots with the taxonomic compositions of the ruminal microbiome in week 0 (merged for all animals in the trial) and in weeks 8 and 16 (merged for all animals within an experimental group). The inner circles represent the composition on phylum level, while the 2nd circles from the inside represent the composition on order level, the 3rd circles from the inside represent the composition on family level and the outer circles represent the genus level. Taxonomic assignments and corresponding colors for each circle are displayed in week 0. B/F resembles the ratio between the main phyla *Bacteroidetes* and *Firmicutes*. CON: groups fed with a control diet, GLY: groups fed with a glyphosate contaminated diet, CFP: concentrate feed proportion, HC: groups with a high CFP, LC: groups fed with a low CFP. The individual subplots including proportions of the taxonomic groups can be found as interactive.htm plots in Supplementary Data 1.

In addition to diversity measures, the taxonomic composition of the rumen microbiota was analyzed. In all experimental groups, the majority of ruminal bacteria were classified as members of the phyla *Bacteroidetes* and *Firmicutes*, while *Tenericutes*, *Actinobacteria*, and *Spirochaetes* also showed relatively high abundances. Rare phyla (<1%) and unclassified reads made up for 3.6% (GLY HC in week 8) to 6.2% (GLY LC in week 8) of the total sequenced reads. At the beginning of the trial, the ratio between *Bacteroidetes* and *Firmicutes* was at 1.5 for the pooled herd. It stayed in a range between 1.3 and 1.5 for the LC groups throughout the trial, while for the HC groups a decrease to values between 0.9 and 1.1 was observed (Fig. 4). The phylum *Bacteroidetes*, was completely assigned to the order *Bacteroidales* and the *Prevotellaceae* were the dominant family within this order and also within the complete microbiome. Average proportions of total classified reads per group that were assigned to the *Prevotellaceae* ranged from 38.7% for the GLY HC group in week 16 to 45.7% for the CON LC group in week 16 (Fig. 4, Supplementary Data 1). The four major genera from this family consisted of uncultured *Prevotella* species. Depending on CFP, especially shifts of *Prevotella* 1, *Prevotella* UCG-001, and *Prevotella* UCG-003 in comparison to *Prevotella* 7 were apparent. While *Prevotella* 7 was associated with a high CFP, the other genera were visually associated with a low CFP. Further *Bacteroidales* belonged to uncultured groups from the *Rikenellaceae*, to the uncultured *Bacteroidales* F082 and RF16 groups or were considered rare taxa (<1% in all conditions tested). Like *Prevotella* 1, these taxa were visually associated with a low CFP (Fig. 4, Supplementary Data 1). The *Firmicutes* were represented by taxa from the order *Clostridiales*, which accounted for 29.4% to 34.7% of total classified reads and the *Erysipelochtrichales*, *Selenomonadales*, and *Lactobacillales*, which each had high abundance (>1%) in at least one group (Fig. 4, Supplementary Data 1). In groups with a high CFP the proportion of *Erysipelochtrichales*, represented by uncultured *Erysiletrichaceae* and *Sharpea*, was strongly increased in comparison to groups with a low CFP (2.2–3.5% in LC; 9.6–12.1% in HC), while the proportion of *Clostridiales* did not show a strong response with respect to the high abundance. Within the *Clostridiales*, however, an enrichment of *Lachnospiraceae* and a reduction of *Christensenellaceae* and *Ruminococcaceae* was observed in response to increased CFP (Fig. 4, Supplementary Data 1). In addition to the changes in the dominant phyla, an increase of *Actinobacteria* was observed with increased CFP (Fig. 4, Supplementary Data 1). Clear changes in the taxonomic composition in relation to glyphosate were not observed.

### Association of microbiome data with glyphosate and CFP

**Fig. 5:**
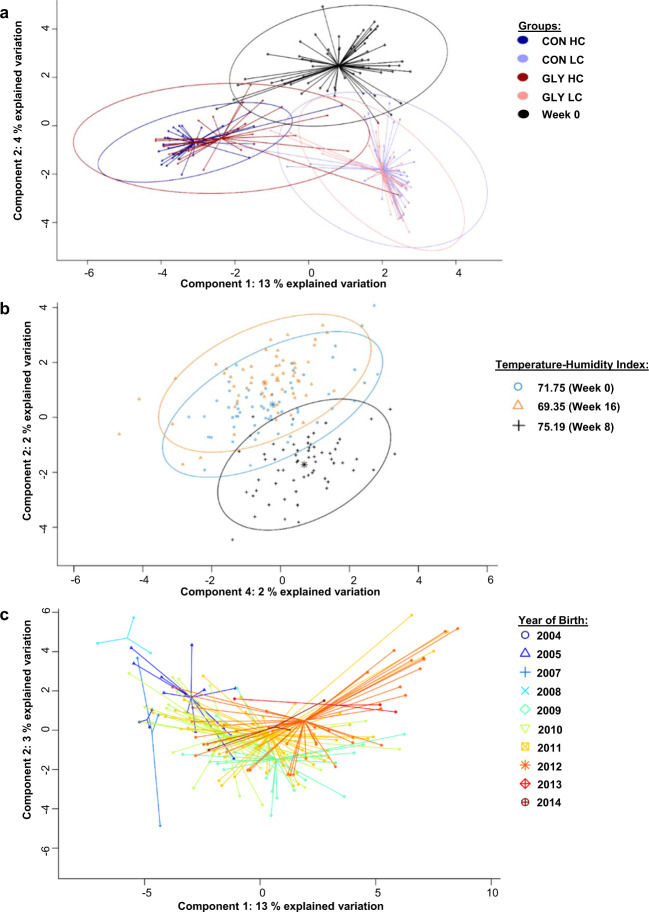
Partial least squares—discriminant analyses of the ruminal microbiome in context of different discrimination factors. Results of three separate PLS-DA analyses using either concentrate feed proportion and glyphosate (**a**), the temperature-humidity-index (**b**), or the year of birth (**c**) as discrimination factors for a dataset containing the microbial taxons of the rumen microbiome. CON: groups fed with a control diet, GLY: groups fed with a glyphosate contaminated diet, CFP: concentrate feed proportion, HC: groups with a high CFP, LC: groups fed with a low CFP.

**Fig. 6:**
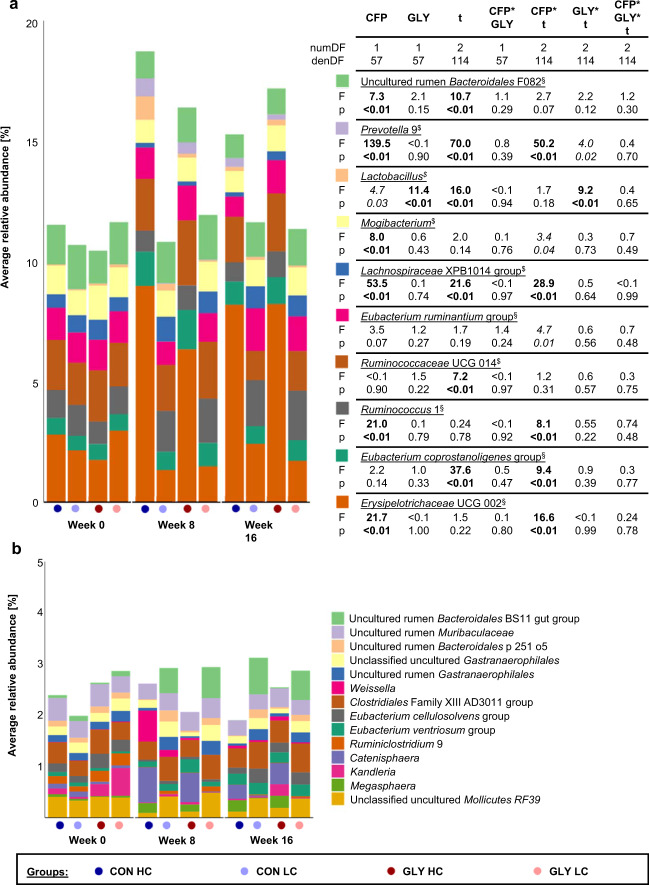
Relative abundance of selected microbial taxons that were relevant for the separation of dairy cows fed with different concentrate feed proportions in the context of glyphosate contaminations in their diets. Relative proportions of the top 10 most abundant taxons (abundance >0.75% in at least one experimental condition) with a VIP score >1 together with influences of the main experimental factors on their abundance as determined by a linear mixed effect model (**a**) and relative proportions of microbial taxons with VIP scores >1 and an abundance >0.2% in at least one experimental condition (**b**). CON: groups fed with a control diet, GLY: groups fed with a glyphosate contaminated diet, CFP: concentrate feed proportion, *t*: time, HC: groups with a high CFP, LC: groups fed with a low CFP, denDF: denominator degrees of freedom, numDF: numerator degrees of freedom, values in bold: *p* < 0.01, values in italics: *p* < 0.05, §: linear spatial covariance structure, $: unconstrained covariance structure.

In order to find microbial taxons that are associated with varying CFPs and glyphosate contaminations in the diet, data of all experimental groups were pooled for week 0, while for weeks 8 and 16 data were pooled according to diet irrespective of time in the trial resulting in groups Week 0, CON HC, CON LC, GLY HC, and GLY LC. The pooled dataset was subjected to PLS-DA and the groups formed three clusters based on the CFP: Week 0 (40% CFP), GLY HC together with CON HC (60% CFP), and GLY LC together with CON LC (30% CFP), while overlaps of GLY groups with their respective CON groups were observed (Fig. 5a). For comparison, separations based on the environmental factor temperature-humidity index (THI, Fig. 5b) or based on the animal-specific factor year of birth (Fig. 5c) could be observed in separate PLS-DA analyses. When having a look at the microbial taxons relevant for separation in the PLS-DA analysis based on CFP and glyphosate contaminations (VIP score>1 in at least one of 9 components, Supplementary Data 2), significant changes in the abundance of 9 out of the top 10 microbial taxons (abundance >0.75% in at least one group) were associated with CFP or changes in abundance over time differed between groups with different CFP (Fig. 6a). *Ruminococcaceae* UCG 14, the remaining genus in the top 10 microbial taxons, showed significant changes over time independent of glyphosate or CFP (p_t_ < 0.01; Fig. 6a). Differing changes over time between GLY and CON groups or significant influences of glyphosate within the top 10 microbial taxons were observed for *Lactobacillus* (p_GLY_ < 0.01; p_GLY*t_ < 0.01) and *Prevotella* 9 (p_GLY*t_ < 0.05; Fig. 6a). When considering further microbial taxons with VIP scores >1 and with an abundance >0.2% in at least one experimental group, 12 out of 14 taxons were significantly influenced by CFP or abundances changed over time in a CFP-dependent manner. Among these CFP-responsive taxons, *Weissella* was influenced by glyphosate (p_GLY _< 0.05) and changes over time differed between CON and GLY groups (P_GLY*t_ < 0.01). The remaining two taxons were not influenced by CFP. *Kandleria* was not influenced by any experimental factor, while the *Eubacterium ventriosum* group was significantly influenced by time and glyphosate (Fig. 6b, Supplementary Table 3). In summary, 21 of 24 microbial taxons relevant for separation in PLS-DA based on experimental factors were significantly influenced by CFP or changes over time were differing based on CFP (Fig. 6a, b, Supplementary Table 3). One taxon was not significantly influenced by any experimental factor and abundances of *Lactobacillus*, *Prevotella* 9, *Weissella*, and the *Eubacterium ventriosum* group were influenced by glyphosate or combinations of glyphosate and further experimental factors (Fig. 6a, b, Supplementary Table 3).

### Association of microbiome data with feed and rumen data

**Fig. 7:**
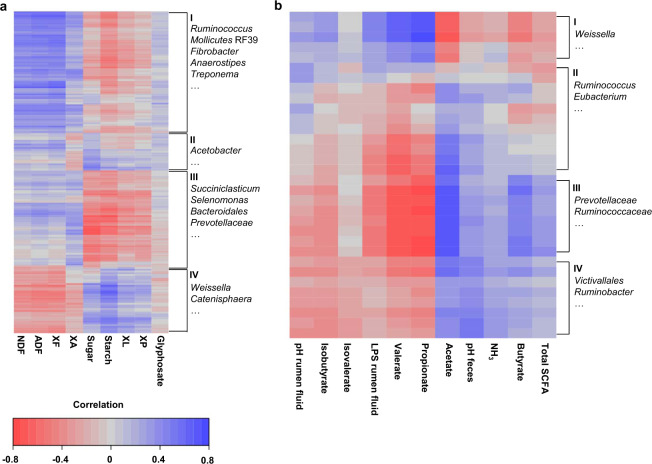
PLS of ruminal microbiota with feed intake and microbial products in the rumen. Clustered image maps of PLS analyses comparing dietary component intake and glyphosate exposure with rumen microbial taxons (**a**) and rumen microbial taxons with microbial products in the rumen and pH of rumen fluid and feces (**b**). NDF: neutral detergent fiber, ADF: acid detergent fiber, XF: crude fiber, XA: crude ash, XL: crude fat, XP: crude protein.

To get further insights, whether exposure to glyphosate and intake of dietary components correlate with abundance of specific rumen microbial taxons and in how far abundance of specific taxons correlates with rumen microbial products, PLS analyses were conducted. The correlation coefficients between glyphosate exposure and abundance of microbial taxons were weak (−0.3 < *r* < 0.3) and did not influence clustering, while correlation coefficients for intake of other feed components led to clustering of ruminal bacterial taxons into four major clusters (Fig. 7a). The first cluster, represented by *Ruminococcus*, *Fibrobacter*, and *Treponema*, showed positive correlations with dietary fiber and mineral intake, while displaying negative correlations with intake of dietary sugar, starch, fat, and protein (Fig. 7a, cluster I). A second smaller cluster, represented by *Acetobacter*, showed correlations with dietary fiber and sugar intake, while negatively correlating with dietary mineral intake (Fig. 7a, cluster II). The third cluster including different *Prevotellaceae* and *Succiniclasticum* was negatively correlated to sugar, starch, fat, and protein intake (Fig. 7a, cluster III) and a fourth cluster, represented by *Weissella* and *Catenisphaera* was negatively correlated to intake of dietary fiber components, while being positively correlated to intake of dietary starch (Fig. 7a, cluster IV). The PLS analysis of rumen microbial products and bacterial taxons in the rumen led to clustering of ruminal microbes into 4 main clusters (Fig. 7b). In general, genera like *Ruminococcus*, that were associated with dietary fiber intake, were positively correlated with ruminal acetate and butyrate proportions, while genera like *Weissella*, positively correlated to dietary starch intake, were also positively correlated to ruminal propionate and valerate proportions (Fig. 7a, b). Interestingly, *Weissella* also displayed a positive correlation to ruminal LPS, while belonging to the Gram-positive bacteria. While correlations of microbial genera with feed component intake and microbial products was relatively strong (*r* up to 0.8 and −0.8), correlations with glyphosate exposure, as mentioned, ruminal pH, pH of feces, and ruminal ammonia were comparably weak (−0.3 ≤ *r* ≤ 0.3).

### Detection of *C. botulinum* neurotoxin gene presence

**Fig. 8:**
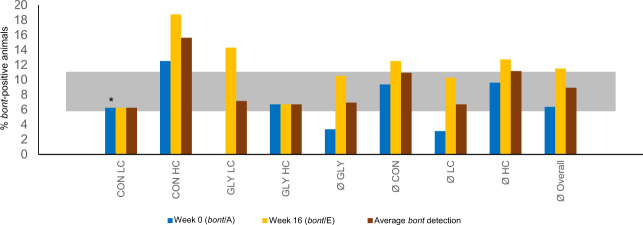
Detection of *C. botulinum* neurotoxin (BoNT) gene presence in fecal samples. Depicted are the proportions of *bont*-positive animals at the beginning of the trial (Week 0, only *bont*/A, PCR detected) and in week 16 (only *bont*/E, real-time-PCR detected). Displayed in gray are the average *bont*-postitive proportions in German cattle herds according to Fohler et al.^36^. CON: groups fed with a control diet, GLY: groups fed with a glyphosate contaminated diet, CFP: concentrate feed proportion, HC: groups with a high CFP, LC: groups fed with a low CFP, *: detection in rumen fluid, average *bont* detection: mean of week 0 and week 16, Ø GLY: mean *bont*-positive animals in GLY groups, Ø CON: mean *bont*-positive animals in CON groups, Ø HC: mean *bont*-positive animals in HC groups, Ø GLY: mean *bont*-positive animals in LC groups, Ø overall: mean *bont*-positive animals without group consideration.

Since precise detection of pathogenic *Clostridia* via 16S rRNA genotyping is not possible due to sequence identities with non-pathogenic *Clostridia*, detection of *C. botulinum* neurotoxin (BoNT) genes in feces and rumen fluid at the beginning and at the end of the trial was used to gain more detailed information about these microorganisms. At the beginning of the trial, before glyphosate exposure, *bont*/A-presence was detected in 6.4% of all animals (*n* = 4), which is within the borders of average detection among German dairy cattle herds (Fig. 8)^36^. These detections were spread among the groups CON LC (*n* = 1), which was the only sample with detection in rumen fluid, CON HC (*n* = 2) and GLY HC (*n* = 1). At the end of the trial in week 16 *bont*/E-presence was detected in 11.5% of all animals (*n* = 7), which is slightly above average detection rate for *bont*-positive animals in German dairy cattle herds according to literature (Fig. 8)^36^. Detection was distributed over feces samples of all groups and detection rate was highest in CON HC (*n* = 3) followed by GLY LC (*n* = 2), CON LC (*n* = 1), and GLY HC (*n* = 1).

In general, numbers of *bont*-positive animals were too low to draw statistically valid conclusions. However, all animals remained healthy throughout the trial and no signs of intestinal neurotoxin-related effects were observed.

## Discussion

Glyphosate has been suspected to have deleterious effects on specific microorganisms in the ruminal microbiome possibly leading to pathogen enrichment and to an inhibited ruminal fiber degradation^27,28^. Moreover, it has been proposed that these effects introduce a dysbiotic state to the ruminal microbiome in interaction with the feed composition^28^. Accordingly, putative disturbances to the rumen microbiome introduced by glyphosate in this study will be discussed in the context of varying CFP and dietary fiber content. On the one hand, possible CFP-related and dietary fiber-related glyphosate effects will be evaluated, while on the other hand the diet-related effects will serve as a comparison and point out the validity of the results of this study. SCFAs, ammonia, pH, and LPS are determinants of microbial metabolism and microbial composition in the rumen, and their concentrations are directly linked to the composition of the diet. High CFP diets are linked to rapid utilization of easy-fermentable carbohydrates by the ruminal microbiome, which can lead to temporary drops in ruminal pH based on acidic fermentation products^38^. These drops can cause subacute ruminal acidosis (SARA), whose susceptibility is varying among individual animals^39^. SARA challenges the ruminal microbiome and can induce lysis of Gram-negative bacteria for example, which in turn is reflected in LPS in the rumen fluid^40,41^. The data in this study suggests increased LPS in rumen fluid in the groups fed with a high CFP, which could be attributed to individual animals with strongly increased LPS concentrations. The absence of changes in ruminal pH in comparison to an expected CFP-induced pH decrease can be explained by the fact that samples were taken in the morning when the cows had a period without fresh feed due to milking and additionally remained without food supply throughout sample collection^10^. Accordingly, rapid drops in pH were not detected. However, pH levels and the number of animals with marked increases in LPS were evenly spread among CON and GLY groups, which indicates that glyphosate had no influence on a putative lysis of ruminal Gram-negative bacteria or ruminal pH in general independent of dietary fiber content or challenge by a high CFP. Remarkably, total ruminal SCFA concentrations seemed to be dropping in the GLY LC group after glyphosate exposure. However, when taking the non-normalized data into account, it becomes apparent that the observed effect is biased by significantly higher total ruminal SCFA concentrations in the group GLY LC (mean 56.2 mmol L^−1^) in comparison to the other groups (pooled mean 45.2 mmol L^−1^) prior to the trial. The stronger decrease in total ruminal SCFA concentrations in this group rather reflects an adjustment to the total SCFA levels of the other groups than a glyphosate effect (mean 35.3 mmol L^−1^ for GLY LC in week 16, mean 34.3 mmol L^−1^ for the pooled other groups in week 16). The proportions of SCFAs were displaying a pattern related to dietary fiber and CFP and were not responsive to glyphosate, which was expected based on studies in sheep and in vitro^8,29^. Isovalerate was seemingly influenced by glyphosate and displayed lower proportions in the GLY groups, which is contradicting with existing literature, where isovalerate proportions were increasing upon glyphosate exposure in an in vitro rumen simulation^29^. The isovalerate proportion was highest for group CON LC in week 16 (average 1.79%), while the other groups had lower proportions (average 1.29%). However, isovalerate has a small proportion among total SCFAs which is linked to changes in the major SCFAs and observed variations are within a range that was observed for animals from this herd independent of experimental conditions previously^42,43^. Accordingly, statistical significances based on these variations are most likely incidental findings and not reproducible. The increased amount of ruminal ammonia in the groups with a low CFP displays the balance of increased crude protein content in the trial diets, differences in urea recycling, and decreased energy supply inhibiting microbial metabolism^10^. It has been proposed that detrimental glyphosate effects on microbiomes rather occur in fiber-rich diets based on in vitro data^28^. However, neither the data of the alpha-diversity nor beta-diversity analyses indicated an effect of glyphosate on microbial diversity in the rumen. In contrast to this, reduced alpha-diversity, evenness, and an increased dominance were related to high CFP. Likewise, in this trial animals fed with an elevated CFP also significantly differed from animals with a low CFP in beta-diversity analyses. The composition of the main taxons in the analyzed rumen microbiomes in this study included *Bacteroidetes, Firmicutes*, *Tenericutes,* and *Spirochaetes* on phylum level and *Prevotella*, *Ruminococcus*, *Butyvibrio*, *Shuttleworthia*, and *Clostridium* on genus level, which is in agreement with microbiome analyses performed on the herd of the animals in this trial previously and with literature on core rumen microbiomes of dairy cattle^44,45^. A decreased proportion of *Bacteroidetes* was associated with increased CFP, the proportion of *Firmicutes* was increased upon high CFP, while no effects of glyphosate were observable. However, a detailed analysis of the data on genus level indicated, that a large proportion of the identified microbial taxons have not been cultured and identification was based on data from previous 16S rDNA genotyping studies of ruminal microbiota. Furthermore, discrepancies between genotype and phenotype have been discussed in the context of ruminal microbiota^44^. Accordingly, it was proposed previously, that characterization of ruminal microbiome should focus on functional properties rather than on genotyping based on 16S rDNA genotyping^44,46^. Despite these challenges related to discrepancies between genotyping and existing phenotypes in the analyzed microbiomes, PLS-DA pointed out a complete overlap of GLY and CON treatment groups based on their CFP. In comparison, seasonal changes in environmental temperature or differences in the year of birth could be detected by PLS-DA. This confirms the general observation, that the ruminal microbiome is shaped by various factors and strongly indicates, that independent of dietary fiber content or challenging conditions for the ruminal microbiome by a high CFP, detrimental influences of glyphosate on the microbiome as a functional unit are most likely negligible in vivo. When considering individual microbial genera, as expected, most relevant microbial taxons responsible for group separation in PLS-DA were statistically associated with the CFP in the diet, while changes related to glyphosate were observed only for 4 taxons. However, when analyzing the abundances outside of the statistical model, it becomes apparent, that glyphosate effects for *Weissella* and *Lactobacillus* should be attributed to an increase of *Lactobacillaceae* in the CON HC group in week 8 rather than specifically to the glyphosate treatment and might reflect short-term drops in ruminal pH^47^. Also, for the taxons from the *Eubacterium ventriosum* group and *Prevotella 9*, glyphosate-based effects are possibly statistical artifacts, as abundances clearly follow comparable CFP-related patterns in GLY and in CON groups. Moreover, a physiological relevance is unlikely due to limited abundance of these taxons. In agreement with observed separation based on CFP in PLS-DA, correlations of microbial taxons with intake of specific feed components and concentrations of specific ruminal metabolites could be observed. For example, *Weissella* abundance was positively correlated to intake of starch and sugar, as well as to the concentrations of propionate, valerate, and LPS in the rumen. *Weissella* belongs to the hetero-fermentative lactic acid bacteria and produces lactic acid and acetate through rapid fermentation^48^. Accordingly, it seems logical that *Weissella* occurs in positive correlation with LPS, since rapid fermentation is linked to rumen acidification and consequently lysis of bacteria, as discussed above. Such CFP-dependent effects were observed for multiple bacterial genera in the rumen and were coherent in the context of rumen metabolism. In contrast, for glyphosate, no coherent effects on bacterial genera could be observed and correlations between glyphosate exposure and abundance of bacterial genera remained elusive (−0.3 < *r* < 0.3). The detection of BoNT gene presence in the fecal and rumen fluid samples was limited to one or two animals per group and sampling and there were no clear indications for influences by CFP or GLY. The proportions of *bont*-positive animals on herd level were reflecting expectations based on literature^36^. As no signs of disease were observed and no increase of *bont*-positive animals was observed despite a highly increased glyphosate exposure in the GLY groups in comparison to the average daily exposure of cattle in Germany, a glyphosate-based effect on pathogenic *Clostridia* can be rejected for this study despite a relatively low number of animals used in the trial.

When summarizing the results of the analyses of rumen microbial products, the composition of the ruminal microbiome, the associations of rumen microbial taxa with feed and microbial products and finally the detection of fecal pathogenic *Clostridia* it becomes obvious, that the principal diet composition unsurprisingly had a strong impact on the ruminal microbiome and its fermentation products, while glyphosate apparently had no relevant effects. This is in agreement with in vitro observations using mixed cultures of microorganisms from ruminal fluid showing that glyphosate had no adverse effects on simulated ruminal microbiomes^29,30^. The absence of long-term glyphosate effects in the gastrointestinal tract is not surprising, since excess of amino acids is available and synthesis of aromatic amino acids can possibly be bypassed through transport^19^. However, it was pointed out recently, that *Salmonella* isolates from before broad usage of glyphosate are more sensitive toward glyphosate than modern isolates^49^. Given that typical ruminal bacteria have been exposed to low concentrations of glyphosate on regular basis, it might be possible that a common ruminal microbiome is adapted to glyphosate exposure. Consequently, under exposure to “real-life” scenario concentrations of glyphosate, effects might not occur in vivo due to previous adaptations, while studies using approaches with highly elevated glyphosate concentrations in vitro could still display effects due to the nature of study design. This, however, is primarily of academic interest, since in reality most cattle herds worldwide would be carrying adapted microbiomes and are not be exposed to highly elevated ruminal glyphosate concentrations used in in vitro trials.

## Methods

### Ethical statement

The experiment was accomplished in accordance with the German Animal Welfare Act approved by the LAVES (Lower Saxony State Office for Consumer Protection and Food Safety, Germany) at the experimental station of the Institute of Animal Nutrition, Friedrich-Loeffler-Institut (FLI), Braunschweig, Germany^10^.

### Experimental feed and set-up

The feed for this trial was produced at the experimental station of the Institute of Animal Nutrition, Friedrich-Loeffler-Institut (FLI), Braunschweig, Germany^10^. In short, subsets of cultivated plants intended for feedstuff production were treated with glyphosate pre-harvest according to the legal regulations. Afterward total mixed rations (TMR) with high (60% CFP, 14% crude fiber (=HC)) and low (30% CFP, 21% crude fiber (=LC)) CFP were prepared as glyphosate contaminated (GLY HC, GLY LC) and glyphosate free (CON HC, CON LC) rations^10^. Sixty-four Holstein cows were assigned to the resulting four groups considering an even distribution of age, performance, and body condition, and 61 of these cows were used for data analyses in the trial^10^. Two cows were removed from the trial due to non-glyphosate-related diseases and suffered from abomasal displacement or general peritonitis. Another cow became dry in trial week 11 and was excluded from the trial^10^. Cows were kept in separated free stall-barns and fed the experimental diets over a period of 16 weeks.

### Sample collection

All rumen fluid samples were collected after milking in the morning at the beginning of the trial and after 8 and 16 weeks of feeding the experimental diets by using the oro-ruminal probe^50^. The procedure included discarding ~200–300 mL of rumen fluid after initial suction before the sample of interest was collected to minimize saliva contamination. After collection, samples for sequencing and measurements of ruminal SCFAs were stored at −20 °C until further processing. Samples for measurements of ammonia and ruminal LPS were centrifuged and heat treated before storage at −80 °C. Further animal parameters were documented as described^10^.

### Measurement of ruminal LPS content

LPS concentration in rumen fluid samples was determined using the *Limulus* amebocyte lysate assay (Kinetic-QCL, Lonza, Walkersville, MD, USA)^51^.

### Measurement of ruminal SCFA and ammonia concentrations

Total SCFA concentrations including acetate (C2%), propionate (C3%), butyrate (C4%), valerate (C5%), isobutyrate (iC4%), and isovalerate (iC5%) were determined as described^52^. Ammonia (NH_3_-N) concentration was determined using steam distillation, according to Kjedahl method DIN38406-E5-2^53^.

### Detection of *C. botulinum* neurotoxin genes

The detection of *C. botulinum* neurotoxin genes and expression in rumen fluid and fecal samples was conducted by PCR-based methods^36^. In short PCR-based detection of *C. botulinum* neurotoxin genes A to F was performed by PCR and supported by real-time-PCR-based detection^54,55^.

### DNA extraction and 16S rDNA sequencing

Rumen fluid samples were thawed at 4 °C overnight and 25 ml sample material was centrifuged for 5 min to remove feed debris and protozoans. Supernatant was collected and processed using a Mikro-Dismembrator S (Sartorius, Goettingen, Germany) for 2 min at a frequency of 2000 min^−1^ in vials cooled by liquid nitrogen. DNA was isolated and purified using the QIAmp DNA Mini Kit (Quiagen, Hilden, Germany; following the manufacturer’s instructions). 16S rDNA was amplified using HotStarTaq DNA polymerase (Qiagen, Hilden, Germany; following the manufacturer’s instructions) and derivatives of primers Com1/Com2-ph with barcode and adapter sequences for IonTorrent PGM (Thermo Fisher Scientific, Waltham, MA, USA, primers see Supplementary Table 4)^56^. After PCR purification using the Min Elute PCR Purifikation Kit (Qiagen, Hilden, Germany) and subsequent quality control, PCR reactions for one sample were pooled and randomly assigned to multiplex Ion PGM sequencing runs using the Ion PGM™ Hi-Q™ View OT2 Kit (Thermo Fisher Scientific, Waltham, MA, USA) for emulsion PCR and the Ion PGM™ Hi-Q™ View Sequencing Kit (Thermo Fisher Scientific, Waltham, MA, USA) for sequencing.

### Data preparation

After adapter removal and demultiplexing were performed with 454 genome sequencer software suite 2.6 (Roche, Mannheim, Germany), sequences were imported into Qiime2^37^. Quality filtering, chimera removal, and denoising were applied by usage of the Dada2 plugin with parameters for trimming 15 nt from the 5′ end and a cutoff length of 265 nt resulting in 250 nt read fragments. Libraries were rarefied to the size of the smallest library.

### Alpha-diversity analysis

Alpha-diversity analysis was conducted using the diversity plugin of Qiime2 using the metrics for the number of observed OTUs, Shannon diversity index, Faith’s phylogenetic diversity index, Pielou evenness, and Berger–Parker dominance^57–60^. Visualizations were conducted using RStudio version 1.1.456 (RStudio Team, 2016) with ggplot2 and ggpubr.

### Beta-diversity analysis

Beta-diversity analysis was conducted using the Qiime2 diversity plugin using weighted UniFrac as distance metric^61^. Group differences were visualized using PCoA and the Qiime2 emperor plugin. Tests for significant group differences were conducted using the Qiime2 plugins adonis, permanova, permdisp, and anosim. Differences were considered significant with *p* < 0.05 and highly significant with *p* < 0.01.

### Taxonomic classification

Taxonomic classification was performed against full-length SILVA SSU 132 Ref NR 99 (release 132) using the consensus-BLAST plugin in Qiime2 with default parameters^62^. Donut plot visualizations of relative proportions were created using RStudio version 1.1.456 (RStudio Team, 2016) with the plotly package.

### Statistical analyses

Unless explicitly stated otherwise, data were collected for each animal in each group (CON HC *n* = 16, CON LC *n* = 16, GLY HC *n* = 15, GLY LC *n* = 14) at every time point (week 0, week 8, and week 16) and considered for the analyses. For statistical analyses of ruminal pH, ammonia concentrations, total ruminal SCFA concentrations, ruminal SCFA proportions, ruminal LPS, alpha-diversity measures, and abundance of rumen microbial taxons, data were normalized for mean values over all groups in week 0 to account for differences before the beginning of the trial and afterward analyzed with linear mixed effect models using RStudio version 1.1.456 (RStudio Team, 2016) with package nlme. Significance was tested via one-tailed ANOVA. Time (*t*; weeks of experiment), treatment (GLY or CON), and CFP (HC or LC diet), as well as their interactions (CFP*t, CFP*GLY, GLY*t, CFP*GLY*t) were applied as fixed factors with random intercepts for individual animals. For each variable, the covariance structure was chosen based on the smallest Akaike information criterion^63^. Effects were considered significant with *p* < 0.05 and highly significant with *p* < 0.01. Relevant rumen microbial taxons for group differences were determined via sPLS-DA using the RStudio version 1.1.456 (RStudio Team, 2016) with package mixOmics^64^. Most suitable number of projection components and microbial taxons per component were calculated using 50-fold validation. Taxons with a VIP score >1 in at least one projection component and with an abundance >0.2% of total reads were considered relevant for detailed analyses. Correlations between feed intake and rumen microbial taxons, as well as between rumen microbial taxons and rumen microbial products were determined using sPLS from the R package mixomics with 50-fold validation. For correlations, a robust Pearson’s-like −0.3 > *r* > 0.3 was considered in order to retain glyphosate exposure in the dataset^65^.

### Reporting summary

Further information on experimental design is available in the Nature Research Reporting Summary linked to this paper.

## Supplementary information

Supplementary Information

Reporting Summary

Supplementary Data 1

Supplementary Data 2

## Data Availability

The sequencing data created for this study has been deposited in the NCBI Sequence Read Archive (SRA) as a bioproject with the accession number PRJNA666764. Further raw data that support the findings of this study are available from the corresponding author upon reasonable request.

## References

[1] Duke SO, Powles SB (2008). Glyphosate: a once-in-a-century herbicide. Pest Manag. Sci..

[2] Dill GM (2005). Glyphosate-resistant crops: history, status and future. Pest Manag. Sci..

[3] Funke T (2006). Molecular basis for the herbicide resistance of Roundup Ready crops. Proc. Natl Acad. Sci. USA.

[4] Dill GM, Cajacob CA, Padgette SR (2008). Glyphosate-resistant crops: adoption, use and future considerations. Pest Manag. Sci..

[5] Steinmann HH, Dickeduisberg M, Theuvsen L (2012). Uses and benefits of glyphosate in German arable farming. Crop Prot..

[6] von Soosten D (2016). Excretion pathways and ruminal disappearance of glyphosate and its degradation product aminomethylphosphonic acid in dairy cows. J. Dairy Sci..

[7] Kubena LF, Smalley HE, Farr FM (1981). Influence of glyphosate (N-(phosphonomethyl)glycine) on performance and selected parameters in broilers. Poult. Sci..

[8] Hüther L, Drebes S, Lebzien P (2005). Effect of glyphosate contaminated feed on rumen fermentation parameters and in sacco degradation of grass hay and corn grain. Arch. Anim. Nutr..

[9] Lee HL (2009). Comparative effects of the formulation of glyphosate-surfactant herbicides on hemodynamics in swine. Clin. Toxicol..

[10] Schnabel K (2017). Effects of glyphosate residues and different concentrate feed proportions on performance, energy metabolism and health characteristics in lactating dairy cows. Arch. Anim. Nutr..

[11] Schnabel K (2020). Functionality and DNA-damage properties of blood cells in lactating cows exposed to glyphosate contaminated feed at different feed energy levels. Arch. Anim. Nutr..

[12] Heymann AK (2021). Effects of glyphosate residues and different concentrate feed proportions in dairy cow rations on hepatic gene expression, liver histology and biochemical blood parameters. PLoS ONE.

[13] Amrhein N, Deus B, Gehrke P, Steinrücken HC (1980). The Site of the Inhibition of the Shikimate Pathway by Glyphosate: II. INTERFERENCE OF GLYPHOSATE WITH CHORISMATE FORMATION IN VIVO AND IN VITRO. Plant Physiol..

[14] Hollander H, Amrhein N (1980). The Site of the Inhibition of the Shikimate Pathway by Glyphosate: I. INHIBITION BY GLYPHOSATE OF PHENYLPROPANOID SYNTHESIS IN BUCKWHEAT (FAGOPYRUM ESCULENTUM MOENCH). Plant Physiol..

[15] Steinrücken HC, Amrhein N (1980). The herbicide glyphosate is a potent inhibitor of 5-enolpyruvyl-shikimic acid-3-phosphate synthase. Biochem. Biophys. Res. Commun..

[16] Borggaard OK, Gimsing AL (2008). Fate of glyphosate in soil and the possibility of leaching to ground and surface waters: a review. Pest Manag. Sci..

[17] Zabaloy MC (2016). Soil ecotoxicity assessment of glyphosate use under field conditions: microbial activity and community structure of Eubacteria and ammonia-oxidising bacteria. Pest Manag. Sci..

[18] Bruckner A (2019). Foliar Roundup application has minor effects on the compositional and functional diversity of soil microorganisms in a short-term greenhouse experiment. Ecotoxicol. Environ. Saf..

[19] Nielsen LN (2018). Glyphosate has limited short-term effects on commensal bacterial community composition in the gut environment due to sufficient aromatic amino acid levels. Environ. Pollut..

[20] Krüger M (2014). Relationship between gastrointestinal dysbiosis and *Clostridium botulinum* in dairy cows. Anaerobe.

[21] Mao SY, Huo WJ, Zhu WY (2016). Microbiome-metabolome analysis reveals unhealthy alterations in the composition and metabolism of ruminal microbiota with increasing dietary grain in a goat model. Environ. Microbiol..

[22] Hua C (2017). Feeding a high concentration diet induces unhealthy alterations in the composition and metabolism of ruminal microbiota and host response in a goat model. Front. Microbiol..

[23] Perea K (2017). Feed efficiency phenotypes in lambs involve changes in ruminal, colonic, and small-intestine-located microbiota. J. Anim. Sci..

[24] Sutton JD (1985). Digestion and absorption of energy substrates in the lactating cow. J. Dairy Sci..

[25] Krause KM, Oetzel GR (2006). Understanding and preventing subacute ruminal acidosis in dairy herds: A review. Anim. Feed Sci. Technol..

[26] Klevenhusen, F. et al. Changes in fibre-adherent and fluid-associated microbial communities and fermentation profiles in the rumen of cattle fed diets differing in hay quality and concentrate amount. *FEMS Microbiol. Ecol.* **93**, 10.1093/femsec/fix100 (2017). 10.1093/femsec/fix100 28922800

[27] Krüger M, Shehata AA, Schrödl W, Rodloff A (2013). Glyphosate suppresses the antagonistic effect of *Enterococcus* spp. on *Clostridium botulinum*. Anaerobe.

[28] Ackermann W (2015). The influence of glyphosate on the microbiota and production of botulinum neurotoxin during ruminal fermentation. Curr. Microbiol..

[29] Riede S (2016). Investigations on the possible impact of a glyphosate-containing herbicide on ruminal metabolism and bacteria in vitro by means of the ‘Rumen Simulation Technique’. J. Appl. Microbiol..

[30] Bote K (2019). Effect of a glyphosate-containing herbicide on *Escherichia coli* and Salmonella Ser. Typhimurium in an in vitro rumen simulation system. Eur. J. Microbiol. Immunol..

[31] Vicini JL, Reeves WR, Swarthout JT, Karberg KA (2019). Glyphosate in livestock: feed residues and animal health1. J. Anim. Sci..

[32] Böhnel H, Schwagerick B, Gessler F (2001). Visceral botulism-a new form of bovine *Clostridium botulinum* toxication. J. Vet. Med. A, Physiol., Pathol., Clin. Med..

[33] Böhnel H, Neufeld B, Gessler F (2005). Botulinum neurotoxin type B in milk from a cow affected by visceral botulism. Vet. J..

[34] Krüger M (2012). Visceral botulism at dairy farms in Schleswig Holstein, Germany: prevalence of *Clostridium botulinum* in feces of cows, in animal feeds, in feces of the farmers, and in house dust. Anaerobe.

[35] Seyboldt C (2015). Occurrence of *Clostridium botulinum* neurotoxin in chronic disease of dairy cows. Vet. Microbiol..

[36] Fohler S (2016). Detection of *Clostridium botulinum* neurotoxin genes (A–F) in dairy farms from Northern Germany using PCR: a case-control study. Anaerobe.

[37] Bolyen E (2019). Reproducible, interactive, scalable and extensible microbiome data science using QIIME 2. Nat. Biotechnol..

[38] Kim Y-H (2018). Changes in ruminal and reticular pH and bacterial communities in Holstein cattle fed a high-grain diet. BMC Vet. Res..

[39] Jing L (2018). Susceptibility of dairy cows to subacute ruminal acidosis is reflected in milk fatty acid proportions, with C18:1 trans-10 as primary and C15:0 and C18:1 trans-11 as secondary indicators. J. Dairy Sci..

[40] Andersen P, Bergelin B, Christensen K (1994). Effect of feeding regimen on concentration of free endotoxin in ruminal fluid of cattle. J. Anim. Sci..

[41] Wells JE, Russell JB (1996). Why do many ruminal bacteria die and lyse so quickly?. J. Dairy Sci..

[42] Schären M (2016). The effects of a ration change from a total mixed ration to pasture on rumen fermentation, volatile fatty acid absorption characteristics, and morphology of dairy cows. J. Dairy Sci..

[43] Schären M (2017). Differential effects of monensin and a blend of essential oils on rumen microbiota composition of transition dairy cows. J. Dairy Sci..

[44] Schären M (2017). Alterations in the rumen liquid-, particle- and epithelium-associated microbiota of dairy cows during the transition from a silage- and concentrate-based ration to pasture in spring. Front. Microbiol..

[45] Xue M (2018). Assessment of rumen microbiota from a large dairy cattle cohort reveals the pan and core bacteriomes contributing to varied phenotypes. Appl. Environ. Microbiol..

[46] Morgavi DP, Kelly WJ, Janssen PH, Attwood GT (2013). Rumen microbial (meta)genomics and its application to ruminant production. Anim.: Int. J. Anim. Biosci..

[47] Asanuma N, Hino T (1997). Tolerance to low pH and lactate production in rumen bacteria. Nihon Chikusan Gakkaiho.

[48] Fusco V (2015). The genus Weissella: taxonomy, ecology and biotechnological potential. Front. Microbiol..

[49] Pöppe J (2019). Minimum inhibitory concentration of glyphosate and a glyphosate-containing herbicide in Salmonella enterica isolates originating from different time periods, hosts, and serovars. Eur. J. Microbiol. Immunol..

[50] Geishauser TJBP (1993). An instrument for collection and transfer of ruminal fluid and for administration of water soluble drugs in adult cattle. The Bovine Practitioner.

[51] Gozho GN (2005). Subacute ruminal acidosis induces ruminal lipopolysaccharide endotoxin release and triggers an inflammatory response. J. Dairy Sci..

[52] Geissler C, Hoffmann M, Hiokel B (1976). Ein Beitrag zur gaschromatographischen Bestimmung flüchtiger Fettsäuren. Arch. f.ür. Tierernaehrung.

[53] Panzel, H. In *Deutsche Einheitsverfahren zur Wasser‐, Abwasser‐und Schlammuntersuchung*. (Beuth and Wiley-VCH, 1998).

[54] Takeshi K (1996). Simple method for detection of *Clostridium botulinum* type A to F neurotoxin genes by ploymerase chain reaction. Microbiol. Immunol..

[55] Kirchner S (2010). Pentaplexed quantitative real-time PCR assay for the simultaneous detection and quantification of botulinum neurotoxin-producing clostridia in food and clinical samples. Appl. Environ. Microbiol..

[56] Schwieger F, Tebbe CC (1998). A new approach to utilize PCR-single-strand-conformation polymorphism for 16S rRNA gene-based microbial community analysis. Appl. Environ. Microbiol..

[57] Shannon CE (1948). A mathematical theory of communication. Bell Syst. Tech. J..

[58] Pielou EC (1966). The measurement of diversity in different types of biological collections. J. Theor. Biol..

[59] Berger WH, Parker FL (1970). Diversity of planktonic foraminifera in deep-sea sediments. Science.

[60] Faith DP (1992). Conservation evaluation and phylogenetic diversity. Biol. Conserv..

[61] Lozupone CA, Hamady M, Kelley ST, Knight R (2007). Quantitative and qualitative beta diversity measures lead to different insights into factors that structure microbial communities. Appl. Environ. Microbiol..

[62] Quast C (2013). The SILVA ribosomal RNA gene database project: improved data processing and web-based tools. Nucleic Acids Res..

[63] Akaike H (1974). A new look at the statistical model identification. IEEE Trans. Autom. Control.

[64] Rohart F, Gautier B, Singh A, Lê Cao K-A (2017). mixOmics: an R package for ‘omics feature selection and multiple data integration. PLOS Computational Biology.

[65] González I, Cao K-AL, Davis MJ, Déjean S (2012). Visualising associations between paired ‘omics’ data sets. BioData Min..

