# Supplementary material for: No hints at glyphosate-induced ruminal dysbiosis in cows
Source: NPJ Biofilms Microbiomes. 2021 Mar 25;7:30. doi: 10.1038/s41522-021-00198-4 (PMC7994389; doi:10.1038/s41522-021-00198-4)
Supplement: Supplementary file 1 — Supplementary Information [file 41522_2021_198_MOESM1_ESM.pdf]

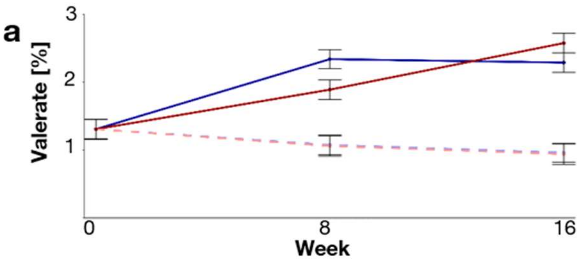

	CFP	GLY	t	CFP* GLY	CFP*t	GLY*t	CFP* GLY*t
F	**78.29**	0.03	**8.2**	<0.01	**28.14**	2.17	2.09
p	**<0.01**	0.85	**<0.01**	0.96	**<0.01**	0.12	0.13

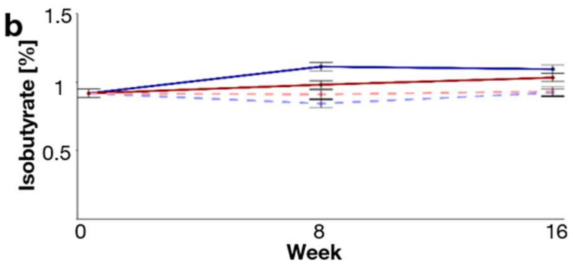

	CFP	GLY	t	CFP* GLY	CFP*t	GLY*t	CFP* GLY*t
F	**21.59**	0.81	**7.26**	4.06	**8.60**	0.33	*3.57*
p	**<0.01**	0.37	**<0.01**	0.05	**<0.01**	0.72	*0.03*

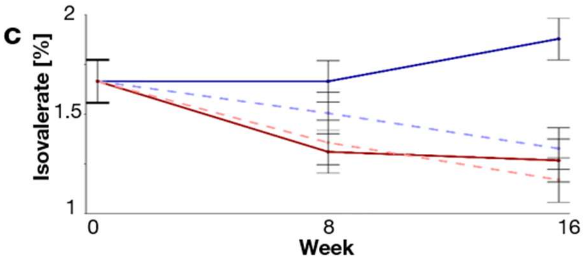

	CFP	GLY	t	CFP* GLY	CFP*t	GLY*t	CFP* GLY*t
F	4.11	*7.17*	**6.85**	2.24	3.13	*3.51*	1.13
p	0.05	*0.01*	**<0.01**	0.14	0.05	*0.03*	0.33

CON HC (n=16)
CON LC (n=16)
GLY HC (n=15)
GLY LC (n=14)

	denDF	numDF
**CFP**	57	1
**GLY**	57	1
**t**	114	2
**CFP* GLY**	57	1
**CFP*t**	114	2
**GLY*t**	114	2
**CFP*GLY*t**	114	2

Supplementary Figure 1: Influence of concentrate feed proportion and glyphosate on minor ruminal SCFAs. Proportions of the minor volatile fatty acids valerate (a), isobutyrate (c) and isovalerate(b). Displayed are lsmeans together with the respective standard error and the results of one tailed ANOVAs of linear-mixed effects models with fixed factors concentrate feed proportion (CFP), glyphosate uptake (GLY) and time (t) and their interactions for the individual parameters. Number of individuals (n) and degrees of freedom were identical for all parameters and displayed in the figure. Blue: control diet (CON), red: glyphosate contaminated diet (GLY), dark solid lines: high CFP (HC), light dashed lines: low CFP (LC), denDF: denominator degrees of freedom, numDF: numerator degrees of freedom, values in bold: p<0.01, values in italics: p<0.05.

Supplementary Table 1: Librarysizes before and after processing with DADA2.

			Remaining reads after:			
		Input reads	Filtering	Denoising	Chimera removal	% remaining reads after DADA2
**CON-LC**		130230	89002	89002	85815	65.9
	Week 0	**202057**	**138041**	**138041**	**128434**	**63.6**
		312960	214477	214477	190952	61.0
		113455	83842	83842	81322	71.7
	Week 8	**146371**	**106586**	**106586**	**101283**	**69.2**
		203528	152086	152086	143934	70.7
		123075	58880	58880	56245	45.7
	Week 16	**189792**	**126668**	**126668**	**118717**	**62.6**
		338789	223976	223976	202518	59.8
**CON-HC**		128836	93285	93285	87412	67.8
	Week 0	**183522**	**129993**	**129993**	**121564**	**66.2**
		334123	240735	240735	221798	66.4
		125606	83837	83837	79883	63.6
	Week 8	**151165**	**112480**	**112480**	**105605**	**69.9**
		203715	159027	159027	144344	70.9
		117541	55088	55088	52023	44.3
	Week 16	**168110**	**120606**	**120606**	**110107**	**65.5**
		294011	192186	192186	164670	56.0
**GLY-LC**		130145	90528	90528	85861	66.0
	Week 0	**199225**	**139429**	**139429**	**128424**	**64.5**
		323254	218286	218286	200940	62.2
		134701	95367	95367	91322	67.8
	Week 8	**159045**	**114598**	**114598**	**108306**	**68.1**
		292569	190081	190081	170398	58.2
		131002	60689	60689	58672	44.8
	Week 16	**206566**	**143850**	**143850**	**133735**	**64.7**
		315628	213397	213397	196799	62.4
**GLY-HC**		117099	53583	53583	50546	43.2
	Week 0	**187830**	**131840**	**131840**	**122784**	**65.4**
		313031	200733	200733	188146	60.1
		129596	90182	90182	87914	67.8
	Week 8	**153213**	**112076**	**112076**	**104306**	**68.1**
		199604	134681	134681	125075	62.7
		127940	92502	92502	83154	65.0
	Week 16	**194991**	**137942**	**137942**	**127236**	**65.3**
		321564	207920	207920	191875	59.7

Mean library sizes for each group and time point together with the biggest (bottom) and smallest (top) libraries based on input reads. Shown are the numbers of raw input reads, of remaining reads after each filtering step and the proportion of reads remaining after quality control.

Supplementary Table 2: Pairwise comparisons of weighted UniFrac distance.

	permANOVA p(pseudo F)	permdisp p(F)	Anosim p(R)
**Week 8**			
**CFP-effect**			
CON HC – CON LC	**<0.01 (12.4)**	0.11 (2.7)	**<0.01 (0.47)**
GLY HC – GLY LC	**<0.01 (6.3)**	0.16 (1.8)	**<0.01 (0.35)**
**GLY-effect**			
CON HC – GLY HC	0.59 (0.8)	0.88(<0.1)	0.92 (-0.40)
CON LC – GLY LC	0.96 (0.2)	0.80 (0.1)	0.93 (-0.05
**Week 8**			
**CFP-effect**			
CON HC – CON LC	**<0.01 (13.9)**	0.57(0.4)	**<0.01 (0.49)**
GLY HC – GLY LC	**<0.01 (8.3)**	0.08 (3.2)	**<0.01 (0.21)**
**GLY-effect**			
CON HC – GLY HC	0.18 (1.54)	0.15(2.1)	0.06 (0.07)
CON LC – GLY LC	0.78 (0.4)	0.38 (0.8)	0.97 (-0.05

Test results for pairwise group-differences and -similarities from anosim-; permANOVA- and permdisp-tests conducted with the diversity plugin of QIIME2 using a distance matrix based on weighted UniFrac distance. The number of samples used for testing were: CON HC (n=16), CON LC (n=16), GLY HC (n=15) GLY LC (n=14). Given are the p-values for the different tests and their respective test-statistics.

Supplementary Table 3: Relative abundance of selected microbial taxons that were relevant for the separation of dairy cows fed with different concentrate feed proportions in the context of glyphosate contaminations in their diets.

		**CFP**	**GLY**	**t**	**CFP* GLY**	**CFP*t**	**GLY*t**	**CFP* GLY*t**
numDF		**1**	**1**	**2**	**1**	**2**	**2**	**2**
denDF		57	57	114	57	114	114	114
Uncultured rumen	F	**183.6**	0.4	0.8	0.4	**76.6**	1.7	0.2
Bacteroidales BS11 gut group[$]	p	**<0.01**	0.54	0.45	0.54	**<0.01**	0.18	0.85
Uncultured rumen	F	**8.3**	1.9	**6.9**	0.3	*4.1*	1.4	0.2
Muribaculaceae[*]	p	**<0.01**	0.17	**<0.01**	0.56	*0.02*	0.26	0.83
Uncultured rumen	F	**106.7**	0.1	**12.5**	0.7	**41.3**	0.8	0.3
Bacteroidales p 251 o5[$]	p	**<0.01**	0.76	**<0.01**	0.40	**<0.01**	0.45	0.74
Unclassified uncultured	F	**12.8**	1.9	*4.3*	2.8	**6.8**	0.7	1.0
Gastranaerophilales[+]	p	**<0.01**	0.17	*0.02*	0.10	**<0.01**	0.49	0.37
Uncultured rumen	F	**44.7**	0.5	**11.5**	0.1	**30.1**	1.8	0,8
Gastranaerophilales[§]	p	**<0.01**	0.47	**<0.01**	0.80	**<0.01**	0.17	0.47
Weissella[$]	F	*5.2*	*5.2*	**24.7**	<0.1	3.1	**5.4**	<0.1
	p	*0.03*	*0.01*	**<0.01**	0.97	0.05	**0.01**	0.99
Clostridiales Family XIII	F	**36.2**	0.7	*3.3*	0.4	**18.6**	1.7	0.5
AD3011 group[§]	p	**<0.01**	0.40	*0.04*	0.54	**<0.01**	0.19	0.64
Eubacterium cellulosolvens	F	*7.4*	0.7	**50.4**	0.5	*4.0*	0.9	1.4
group[§]	p	*0.01*	0.39	**<0.01**	0.50	*0.02*	0.40	0.25
Eubacterium ventriosum	F	0.3	*5.9*	**29.3**	0.2	2.1	2.6	1.2
group[$]	p	0.58	*0.02*	**<0.01**	0.65	0.13	0.07	0.30
Ruminoclostridium 9[$]	F	**24.8**	1.3	**42.5**	<0.1	**20.4**	1.1	<0.1
	p	**<0.01**	0.25	**<0.01**	0.87	**<0.01**	0.34	0.97
Catenisphaera[+]	F	**41.9**	<0.1	**18.7**	0.2	**61.8**	0.9	0.5
	p	**<0.01**	0.89	**<0.01**	0.62	**<0.01**	0.41	0.63
Kandleria[$]	F	0.5	0.1	2.2	0.7	0.7	0.4	0.4
	p	0.47	0.82	0.11	0.41	0.52	0.65	0.68
Megasphaera[$]	F	**86.9**	<0.1	**6.1**	0.5	**35.8**	0.4	0.2
	p	**<0.01**	0.94	**<0.01**	0.48	**<0.01**	0.68	0.81
Unclassified uncultured	F	**53.7**	<0.1	**22.1**	0.3	**36.4**	<0.1	0.3
Mollicutes RF39[§]	p	**<0.01**	0.91	**<0.01**	0.58	**<0.01**	0.99	0.72

Influences of the main experimental factors CFP, GLY and time on the abundance of selected rumen microbial taxons (0.2% < abundance<0.75 % in at least one experimental condition; VIP score >1 in a PLS-DA analysis with the main experimental factors) determined by linear mixed effect models. §=linear spatial covariance structure, + = gaussian covariance structure, $= unconstrained covariance structure, *= spherical spatial covariance structure, denDF: denominator degrees of freedom, numDF: numerator degrees of freedom, values in bold: p<0.01, values in italics: p<0.05.

Supplementary Table 4: Primers used for this study

Primer	Adapter name	Adapter	Additional	MID-Barcode	Additional	Target
**Universal**						
Com1fw						
ITA2rev_IDxx_Com1fw	trP1	CCTCTCTATGGGCAGTCGGTGAT	CAG	---	---	CAGCAGCCGCGGTAATAC
Com2-Ph rv						
ITA1rev_IDxx_Com2-Ph rv	trP1	CCTCTCTATGGGCAGTCGGTGAT	---	---	---	CCGTCAATTCCTTTGAGTTT
**Barcode**						
Com1fw						
ITA1fwd_ID94_Com1fw	A	CCATCTCATCCCTGCGTGTCTCCGACTCAG	---	TCCGACAAGC	GAT	CAGCAGCCGCGGTAATAC
ITA1fwd_ID93_Com1fw	A	CCATCTCATCCCTGCGTGTCTCCGACTCAG	---	CTTGTCCAATC	GAT	CAGCAGCCGCGGTAATAC
ITA1fwd_ID92_Com1fw	A	CCATCTCATCCCTGCGTGTCTCCGACTCAG	---	CTAGGAACCGC	GAT	CAGCAGCCGCGGTAATAC
ITA1fwd_ID91_Com1fw	A	CCATCTCATCCCTGCGTGTCTCCGACTCAG	---	CGGAAGGATGC	GAT	CAGCAGCCGCGGTAATAC
ITA1fwd_ID90_Com1fw	A	CCATCTCATCCCTGCGTGTCTCCGACTCAG	---	CTAACCACGGC	GAT	CAGCAGCCGCGGTAATAC
ITA1fwd_ID89_Com1fw	A	CCATCTCATCCCTGCGTGTCTCCGACTCAG	---	TCCTGAATCTC	GAT	CAGCAGCCGCGGTAATAC
ITA1fwd_ID88_Com1fw	A	CCATCTCATCCCTGCGTGTCTCCGACTCAG	---	CCGAACACTTC	GAT	CAGCAGCCGCGGTAATAC
ITA1fwd_ID87_Com1fw	A	CCATCTCATCCCTGCGTGTCTCCGACTCAG	---	TTGGCTGGAC	GAT	CAGCAGCCGCGGTAATAC
ITA1fwd_ID86_Com1fw	A	CCATCTCATCCCTGCGTGTCTCCGACTCAG	---	CTTGGTTATTC	GAT	CAGCAGCCGCGGTAATAC
ITA1fwd_ID85_Com1fw	A	CCATCTCATCCCTGCGTGTCTCCGACTCAG	---	CCAGCCTCAAC	GAT	CAGCAGCCGCGGTAATAC
ITA1fwd_ID84_Com1fw	A	CCATCTCATCCCTGCGTGTCTCCGACTCAG	---	CTTCCATAAC	GAT	CAGCAGCCGCGGTAATAC
ITA1fwd_ID83_Com1fw	A	CCATCTCATCCCTGCGTGTCTCCGACTCAG	---	CTAGGACATTC	GAT	CAGCAGCCGCGGTAATAC
ITA1fwd_ID82_Com1fw	A	CCATCTCATCCCTGCGTGTCTCCGACTCAG	---	TTGGCATCTC	GAT	CAGCAGCCGCGGTAATAC
ITA1fwd_ID81_Com1fw	A	CCATCTCATCCCTGCGTGTCTCCGACTCAG	---	CCTGCCATTCGC	GAT	CAGCAGCCGCGGTAATAC
ITA1fwd_ID80_Com1fw	A	CCATCTCATCCCTGCGTGTCTCCGACTCAG	---	TCGAAGGCAGGC	GAT	CAGCAGCCGCGGTAATAC
ITA1fwd_ID79_Com1fw	A	CCATCTCATCCCTGCGTGTCTCCGACTCAG	---	CCTGGTTGTC	GAT	CAGCAGCCGCGGTAATAC
ITA1fwd_ID78_Com1fw	A	CCATCTCATCCCTGCGTGTCTCCGACTCAG	---	CAGCCAATTCTC	GAT	CAGCAGCCGCGGTAATAC
ITA1fwd_ID77_Com1fw	A	CCATCTCATCCCTGCGTGTCTCCGACTCAG	---	CGAAGCGATTC	GAT	CAGCAGCCGCGGTAATAC
ITA1fwd_ID76_Com1fw	A	CCATCTCATCCCTGCGTGTCTCCGACTCAG	---	CGGAAGAACCTC	GAT	CAGCAGCCGCGGTAATAC
ITA1fwd_ID75_Com1fw	A	CCATCTCATCCCTGCGTGTCTCCGACTCAG	---	TCAGGAATAC	GAT	CAGCAGCCGCGGTAATAC

Primer	Adapter name	Adapter	Additional	MID-Barcode	Additional	Target
Com2-Ph rv						
ITA2fwd_ID94_Com2-Ph rv	A	CCATCTCATCCCTGCGTGTCTCCGACTCAG	---	TCCGACAAGC	GAT	CCGTCAATTCCTTTGAGTTT
ITA2fwd_ID93_Com2-Ph rv	A	CCATCTCATCCCTGCGTGTCTCCGACTCAG	---	CTTGTCCAATC	GAT	CCGTCAATTCCTTTGAGTTT
ITA2fwd_ID92_Com2-Ph rv	A	CCATCTCATCCCTGCGTGTCTCCGACTCAG	---	CTAGGAACCGC	GAT	CCGTCAATTCCTTTGAGTTT
ITA2fwd_ID91_Com2-Ph rv	A	CCATCTCATCCCTGCGTGTCTCCGACTCAG	---	CGGAAGGATGC	GAT	CCGTCAATTCCTTTGAGTTT
ITA2fwd_ID90_Com2-Ph rv	A	CCATCTCATCCCTGCGTGTCTCCGACTCAG	---	CTAACCACGGC	GAT	CCGTCAATTCCTTTGAGTTT
ITA2fwd_ID89_Com2-Ph rv	A	CCATCTCATCCCTGCGTGTCTCCGACTCAG	---	TCCTGAATCTC	GAT	CCGTCAATTCCTTTGAGTTT
ITA2fwd_ID88_Com2-Ph rv	A	CCATCTCATCCCTGCGTGTCTCCGACTCAG	---	CCGAACACTTC	GAT	CCGTCAATTCCTTTGAGTTT
ITA2fwd_ID87_Com2-Ph rv	A	CCATCTCATCCCTGCGTGTCTCCGACTCAG	---	TTGGCTGGAC	GAT	CCGTCAATTCCTTTGAGTTT
ITA2fwd_ID86_Com2-Ph rv	A	CCATCTCATCCCTGCGTGTCTCCGACTCAG	---	CTTGGTTATTC	GAT	CCGTCAATTCCTTTGAGTTT
ITA2fwd_ID85_Com2-Ph rv	A	CCATCTCATCCCTGCGTGTCTCCGACTCAG	---	CCAGCCTCAAC	GAT	CCGTCAATTCCTTTGAGTTT
ITA2fwd_ID84_Com2-Ph rv	A	CCATCTCATCCCTGCGTGTCTCCGACTCAG	---	CTTCCATAAC	GAT	CCGTCAATTCCTTTGAGTTT
ITA2fwd_ID83_Com2-Ph rv	A	CCATCTCATCCCTGCGTGTCTCCGACTCAG	---	CTAGGACATTC	GAT	CCGTCAATTCCTTTGAGTTT
ITA2fwd_ID82_Com2-Ph rv	A	CCATCTCATCCCTGCGTGTCTCCGACTCAG	---	TTGGCATCTC	GAT	CCGTCAATTCCTTTGAGTTT
ITA2fwd_ID81_Com2-Ph rv	A	CCATCTCATCCCTGCGTGTCTCCGACTCAG	---	CCTGCCATTCGC	GAT	CCGTCAATTCCTTTGAGTTT
ITA2fwd_ID80_Com2-Ph rv	A	CCATCTCATCCCTGCGTGTCTCCGACTCAG	---	TCGAAGGCAGGC	GAT	CCGTCAATTCCTTTGAGTTT
ITA2fwd_ID79_Com2-Ph rv	A	CCATCTCATCCCTGCGTGTCTCCGACTCAG	---	CCTGGTTGTC	GAT	CCGTCAATTCCTTTGAGTTT
ITA2fwd_ID78_Com2-Ph rv	A	CCATCTCATCCCTGCGTGTCTCCGACTCAG	---	CAGCCAATTCTC	GAT	CCGTCAATTCCTTTGAGTTT
ITA2fwd_ID77_Com2-Ph rv	A	CCATCTCATCCCTGCGTGTCTCCGACTCAG	---	CGAAGCGATTC	GAT	CCGTCAATTCCTTTGAGTTT
ITA2fwd_ID76_Com2-Ph rv	A	CCATCTCATCCCTGCGTGTCTCCGACTCAG	---	CGGAAGAACCTC	GAT	CCGTCAATTCCTTTGAGTTT
ITA2fwd_ID75_Com2-Ph rv	A	CCATCTCATCCCTGCGTGTCTCCGACTCAG	---	TCAGGAATAC	GAT	CCGTCAATTCCTTTGAGTTT
