# Supplementary material for: No hints at glyphosate-induced ruminal dysbiosis in cows
Source: NPJ Biofilms Microbiomes. 2021 Mar 25;7:30. doi: 10.1038/s41522-021-00198-4 (PMC7994389; doi:10.1038/s41522-021-00198-4)
Supplement: Supplementary file 2 — Reporting Summary [file 41522_2021_198_MOESM2_ESM.pdf]

nature research

Corresponding author(s): Fabian Billenkamp

Last updated by author(s): Sep 28, 2020

Reporting Summary

Nature Research wishes to improve the reproducibility of the work that we publish. This form provides structure for consistency and transparency in reporting. For further information on Nature Research policies, see our Editorial Policies and the Editorial Policy Checklist.

Statistics

For all statistical analyses, confirm that the following items are present in the figure legend, table legend, main text, or Methods section.

n/a	Confirmed	
☐	☒	The exact sample size (*n*) for each experimental group/condition, given as a discrete number and unit of measurement
☐	☒	A statement on whether measurements were taken from distinct samples or whether the same sample was measured repeatedly
☐	☒	The statistical test(s) used AND whether they are one- or two-sided<br>*Only common tests should be described solely by name; describe more complex techniques in the Methods section.*
☐	☒	A description of all covariates tested
☒	☐	A description of any assumptions or corrections, such as tests of normality and adjustment for multiple comparisons
☐	☒	A full description of the statistical parameters including central tendency (e.g. means) or other basic estimates (e.g. regression coefficient) AND variation (e.g. standard deviation) or associated estimates of uncertainty (e.g. confidence intervals)
☐	☒	For null hypothesis testing, the test statistic (e.g. *F*, *t*, *r*) with confidence intervals, effect sizes, degrees of freedom and *P* value noted<br>*Give P values as exact values whenever suitable.*
☒	☐	For Bayesian analysis, information on the choice of priors and Markov chain Monte Carlo settings
☒	☐	For hierarchical and complex designs, identification of the appropriate level for tests and full reporting of outcomes
☒	☐	Estimates of effect sizes (e.g. Cohen's *d*, Pearson's *r*), indicating how they were calculated

Our web collection on statistics for biologists contains articles on many of the points above.

Software and code

Policy information about availability of computer code

Data collection	Preparation of NGS libraries: 454 GENOME SEQUENCER SOFTWARE SUITE 2.6, QIIME2(DADA2)
Data analysis	16S rDNA Genotyping: QIIME2 (Diversity, Taxonomic classification), SILVA SSU 132 REF NR 99 database, R (PLOTLY, MIXOMICS),<br>Mixed Models: R(NLME)

For manuscripts utilizing custom algorithms or software that are central to the research but not yet described in published literature, software must be made available to editors and reviewers. We strongly encourage code deposition in a community repository (e.g. GitHub). See the Nature Research guidelines for submitting code & software for further information.

Data

Policy information about availability of data

All manuscripts must include a data availability statement. This statement should provide the following information, where applicable:

- Accession codes, unique identifiers, or web links for publicly available datasets
- A list of figures that have associated raw data
- A description of any restrictions on data availability

The sequencing data created for this study has been deposited in the NCBI Sequence Read Archive (SRA) as a bioproject with the accession number [will be made available upon publication]. Further raw data that support the findings of this study are available from the corresponding author upon reasonable request.

Field-specific reporting

Please select the one below that is the best fit for your research. If you are not sure, read the appropriate sections before making your selection.

☒ Life sciences ☐ Behavioural & social sciences ☐ Ecological, evolutionary & environmental sciences

For a reference copy of the document with all sections, see nature.com/documents/nr-reporting-summary-flat.pdf

Life sciences study design

All studies must disclose on these points even when the disclosure is negative.

Sample size	61 (64) cows (including 16 heifers) in a 2x2 factorial design. Sample size was considdered based on maximum size for trials with dairy cows in the experimental barn and is in agreement with prior studies.
Data exclusions	Two cows were excluded because of diseases not related to the experimental treatments. In the first week of the trial, one cow of group GLHC had an abomasal displacement. A cow of Group GLLC developed a general peritonitis in week 8. Another cow of Group GLLC became dry in trial week 11 and was excluded from the trial. Schnabel (2017).
Replication	Technical replicates of measurements were collected where suitable.
Randomization	48 cows and 16 heifers were assigned to four different feeding groups by considering number of lactation (2.8 ± 0.7) and data that had been collected prior to the trial, presenting a 3-d mean of body weight (BW, 645 ± 21 kg), daily feed intake (40.9 ± 0.2 kg fresh matter of the ration) and fat corrected milk (FCM, 4% fat; 29.1 ± 0.5 kg).
Blinding	Blinding was not possible during the experimental trial. For the acquisition of data in this study, however, blinding was no major concern, since data from all animals that stayed in trial was analyzed according to the same SOPs.

Reporting for specific materials, systems and methods

We require information from authors about some types of materials, experimental systems and methods used in many studies. Here, indicate whether each material, system or method listed is relevant to your study. If you are not sure if a list item applies to your research, read the appropriate section before selecting a response.

Materials & experimental systems

n/a	Involved in the study	
☒	☐	Antibodies
☒	☐	Eukaryotic cell lines
☒	☐	Palaeontology and archaeology
☐	☒	Animals and other organisms
☒	☐	Human research participants
☒	☐	Clinical data
☒	☐	Dual use research of concern

Methods

n/a	Involved in the study	
☒	☐	ChIP-seq
☒	☐	Flow cytometry
☒	☐	MRI-based neuroimaging

Animals and other organisms

Policy information about studies involving animals; ARRIVE guidelines recommended for reporting animal research

Laboratory animals	The study invloved dairy cows and experiment was accomplished in accordance with the German Animal Welfare Act approved by the LAVES (Lower Saxony State Office for Consumer Protection and Food Safety, Germany).
Wild animals	*Provide details on animals observed in or captured in the field; report species, sex and age where possible. Describe how animals were caught and transported and what happened to captive animals after the study (if killed, explain why and describe method; if released, say where and when) OR state that the study did not involve wild animals.*
Field-collected samples	*For laboratory work with field-collected samples, describe all relevant parameters such as housing, maintenance, temperature, photoperiod and end-of-experiment protocol OR state that the study did not involve samples collected from the field.*
Ethics oversight	LAVES (Lower Saxony State Office for Consumer Protection and Food Safety, Germany)

Note that full information on the approval of the study protocol must also be provided in the manuscript.
