# Supplementary material for: No hints at glyphosate-induced ruminal dysbiosis in cows
Source: NPJ Biofilms Microbiomes. 2021 Mar 25;7:30. doi: 10.1038/s41522-021-00198-4 (PMC7994389; doi:10.1038/s41522-021-00198-4)
Supplement: Supplementary file 3 — Supplementary Data 1 [file 41522_2021_198_MOESM3_ESM.zip › Supplementary Data 1/data/Week 16 GLY LC.htm]

Week 16 GLY LC
