# Supplementary material for: No hints at glyphosate-induced ruminal dysbiosis in cows
Source: NPJ Biofilms Microbiomes. 2021 Mar 25;7:30. doi: 10.1038/s41522-021-00198-4 (PMC7994389; doi:10.1038/s41522-021-00198-4)
Supplement: Supplementary file 3 — Supplementary Data 1 [file 41522_2021_198_MOESM3_ESM.zip › Supplementary Data 1/Supplementary Data 1.htm]

# Single plots used in Figure 4

The data in the linked .htm files is created with plotly and uses scripts/ActiveX accordingly.  
Hovering will display proportions of taxonomic groups.

**Taxonomic composition of the ruminal microbiome in response to varying concentrate feed proportion and glyphosate**  
Depicted are donut plots with the taxonomic compositions of the ruminal microbiome in week 0 (merged for all animals in the trial) and in weeks 8 and 16 (merged for all animals within an experimental group). The inner circles represent the composition on phylum level, while the 2nd circles from the inside represent the composition on order level, the 3rd circles from the inside represent the composition on family level and the outer circles represent the genus level. CON = groups fed with a control diet, GLY = groups fed with a glyphosate contaminated diet, CFP = concentrate feed proportion, HC= groups with a high CFP, LC = groups fed with a low CFP.
The data in the linked .htm files is created with plotly and uses scripts/ActiveX accordingly.  
Hovering will display proportions of taxonomic groups.

**Week 0**   
Merged data of all groups in week 0.

**Week 8**   
Week 8 CON HC  
Week 8 CON LC  
Week 8 GLY HC  
Week 8 GLY LC

**Week 16**   
Week 16 CON HC  
Week 16 CON LC  
Week 16 GLY HC  
Week 16 GLY LC
